# A Bogotana House of the Spirits: Venezuelan and Colombian HIV/AIDS Biosociality Beyond-the-Living

**DOI:** 10.1080/01459740.2025.2527097

**Published:** 2025-07-07

**Authors:** Rebecca Irons

**Affiliations:** University College London, London, United Kingdom

**Keywords:** Colombia, Venezuela, biosociality, HIV/AIDS, migration

## Abstract

Biosociality is often referenced regarding those living with HIV/AIDS, but scholarship takes for granted that these relationships exist between living beings. Based on long-term ethnography in refuge houses for Venezuelan migrants and Colombians living with HIV in Bogotá, Colombia, I argue that HIV/AIDS biosociality may also exist between the living and beyond-the-living *espíritus* who historically lived and died with HIV/AIDS. The discussion will show how consideration of biosociality beyond-the-living can act as an important analytical tool in understanding the experiences of migrants living with HIV in Latin America and how they come to interpret their situation and futures.

In the darkness of the crisp Andean winter night, Clara was startled by a woman carefully crossing the second-floor landing, unwavering as she moved from one of the communal bedrooms toward an empty staircase that connected the four floors of the courtyard-style colonial house. Somehow internally illuminated, the woman made no sound at all with her footsteps, seemingly floating across the wooden floor. Clara heard only her own breathing as she observed in silence. She had arrived at the Santa Chiquinquirá refuge house 3 days before, accompanied by her four children and husband. Santa Chiquinquirá is a non-governmental foundation with two houses of refuge for people living with HIV in Bogotá, and shelters those in need free of charge whilst they seek medication and find their feet, regardless of nationality or citizenship status. Whilst it has been predominately utilized by Colombians throughout its 40-year history, in the last decade the refuge has seen a sharp increase of Venezuelan guests as the social, political, and economic crisis of Venezuela has worn on, resulting in the almost eight million migrants that have now left the country by 2024.

Clara and her family had crossed from their home in Maracaibo, Venezuela, to Bucaramanga, Colombia, on foot in 2022, a journey that took them 2 weeks in her condition. She had been pregnant with twins when they left their Caribbean home. Clara gave birth in Bucaramanga but only one of the twins survived, an incident that she was understandably still distraught about when we met in 2023. Her unsupportive husband, Esteban, was busy womanizing the locals during their Bucaramanga stay with Clara’s cousin, and she did not see him very much during the first few months of their new son’s life. When Esteban, and then Clara, was diagnosed with HIV in 2023, the cousin kicked them out over a misplaced panic that they would pass the virus to her children, Clara said. With nowhere else to go, they made their way to Bogotá where they had heard of the Santa Chiquinquirá HIV refuge house through another NGO in Bucaramanga. In June 2023, they joined the other five Venezuelans and one Colombian that were living there at the time. However, on that chilly night early in their stay, the woman that Clara saw was not one of the other guests she had been previously introduced to. From her vantage point on the fourth-floor internal balcony, Clara could watch quietly and removed from this nocturnal visit, as the woman all but disappeared into the shadows. She recounted the story to me over a strong Colombian *tinto* coffee, sweetened with thick *panela*. When I asked her who she thought this person could possibly be, Clara directed me toward a worn photograph inside a dusty plastic frame, hung alongside other images of guests-gone-by, displayed beneath one of the many religious paintings depicting biblical scenes. Here, it was this woman, she stated, nonchalant. She believed the woman in the photo to have been dead some 30 years, but now, was welcoming Clara’s family to Bogotá.

Clara’s experience in Santa Chiquinquirá was not an isolated event. As I will explore in this article, sightings and interactions by/between living individuals with HIV, with now-deceased people who had been living with HIV, are a common occurrence in this refuge house network. I propose taking seriously communication between the living and the beyond-the-living *espíritus* in a context of HIV amongst marginalized communities. As such, I will introduce and discuss the possibility of a biosociality beyond-the-living. Relationships that cross the living/non-living divide are commonly acknowledged in Latin America, where “death is understood as a trajectory that connects ancestral times to current and future times through encounters of living humans with the spirits and souls of deceased humans” (Klaufus 2019:282), and where “people firmly believe they can communicate with the dead in the hereafter,” for the dead are “living agents” (2019:282). In the context of HIV/AIDS, these connections and encounters between living and [beyond-the] “living agents” take on a biosocial nature.

Biosociality may be seen as a concept where people who share biological conditions, here HIV/AIDS, form supportive social networks and collectives based on shared experience (Rabinow and Rose 2006), which could include experiences of stigma-based violence and social isolation. Biosociality has been used to describe and discuss the shared experiences and social collectivity of many groups of individuals uniting over shared biology, with particular emphasis on those living with HIV/AIDS, from Africa (Marsland 2012; Singer 2011), to Asia (Hegarty 2021), Europe (Young 2022), Oceania (Petersen et al. 2019), and Latin America (Valle 2015). However, to my knowledge, all discussions of biosociality focus on relationships between the living.

In this article, I discuss the experience of biosociality between socially marginalized Colombians and, more recently, Venezuelans, who lived with HIV/AIDS and died with HIV/AIDS and now reside in the Santa Chiquinquirá refuges as spirits (*espíritus)*, alongside living Colombians and Venezuelans who are living with HIV in the refuges. Interactions with the dead are not uncommon in the world, and in Latin America especially, however they are rarely taken seriously as objective events. This may be considered a Global North perspective (Baker and Bader 2014). Alternatively, an acceptance of everyday extraordinary events, such as interactions beyond-the-living, may be considered a decolonial approach, invariably because it rests on the objective acceptance of an “irreducible element” that cannot be explained by Western, scientific discourse (Faris 2002:102). This is the approach that I take here.

Broadly speaking, this article is about haunted houses and the relationships between the living and dead in those places. In their extensive research on Latin American deathscapes, Klaufus highlights cemeteries as key *cultural hotspots*, Bogotá in particular, as places where beliefs about life and death are expressed and heightened (2019:278). In particular, the memory politics of cemeteries are thought to speak of violent histories. Due to the complicated nature of the AIDS corpse and social margin-alization (Troyer 2010), the discussion here does not and cannot revolve around a cemetery *per se*, but instead I approach the refuges themselves as *biosocial hotspots* that reflect ideas and beliefs about what it means to live with HIV as a socially marginalized population.

The *espíritus* of Santa Chiquinquirá are ghosts of place, they have only been sighted within the refuge house network, and this says something particular about them. As Callard writes, “ghosts of place – singly haunting a house […] are apparitions governed by history” (Callard 2022:111), here the history of not only the house(s) but of the wider landscape of HIV/AIDS and LGBTQ-directed violence in a nation and time period suffering extreme violence.

On the haunted house genre of haunting, Miller suggests that ghosts be seen as “estate agents,” their presence providing “a resolution to a problem of social and material relations” (Miller 2021:6) in a context whereby a property may be considered fixed, but its inhabitants are always transient. Callard also links ghostly apparitions to earthly material goods, where the ghosts of deceased relatives hold “authority as arbiters in mattes of inheritance” when the record is unclear (2022:120). I suggest that there is an inheritance to be shared between historical residents and present-day residents in Santa Chiquinquirá, but it is not a material one. Instead, the inheritance is a shared narrative of historical and contemporary social stigma and marginalization, of necropower (Mbembe 2003) that may result in the deaths of refuge residents. In the past, this was directed toward Colombians living with [untreatable] HIV/AIDS, most likely members of the LGBTQ community, who moved to the refuges for palliative care. Nowadays, this is directed toward Venezuelan migrants living with HIV, often members of the LGBTQ community but not always, but likely experiencers of xenophobia and racism due to the mass migrant crisis in the Americas (Irons 2022), who have moved to the refuge because they have nowhere else to go. By virtue of having to live in an HIV/AIDS refuge, the residents may be estranged from their families – or their families may be far away in hometowns of Venezuela. Either way, there are limited witnesses to tell the stories of marginalized migrants and even fewer to share the lessons of the dead, and so I suggest that some of them seemingly communicate it for themselves, to each other.

In order to address these themes, I will first introduce the Venezuelan crisis and HIV, exploring how and why contemporary residents come to be in the refuge houses, before discussing the role of ghosts and relationships with the dead in Latin America and the wider literature. Having done so, it will be possible to explore the violent, stigmatizing history of the refuges, and how this impacts present-day residents. I will do this by reviewing the case of an attack, and the question of space-usage in changing times. Finally, I will focus on bilateral events between the living and beyond-the-living, to show how the *espíritus* are biosocially involved in the life of the houses. In so doing, it will be possible to show how biosociality beyond-the-living can be considered an important analytical tool in understanding the experiences of migrants living with HIV and how they come to interpret their situation and futures, as well as the historic experiences of those who lived HIV stigma during times of violence.

## Background

Venezuela is undergoing one of the world’s worst displacement crises and has now become the largest migration emergency in the history of the Americas (UNHCR 2024). This situation is due to the social, political, and economic mismanagement that has intensified in recent years with international trade restrictions. Inevitably, the situation has become extremely difficult for all, not least those citizens living with HIV/AIDS and in-need of anti-retroviral therapy (ART). With the collapse of the public health system, HIV/AIDS-related services and treatments have become reduced dramatically and are often absent across Venezuela (Page et al. 2019, Rendon et al. 2018; Pierce 2017). As of 2019, there were an estimated 8,000 Venezuelans living with HIV outside of Venezuela (UNAIDS 2021). Seventynine thousand four hundred and sixty-seven Venezuelans are estimated to be living with HIV in Venezuela as of 2019 (Schreiber 2019). However, as noted, it is difficult to know exact figures due to a lack of official reporting. When data were last available, from 2010 to 2016, a 24 percent increase in HIV diagnosis was reported by PAHO (2018). This was due to a lack of ART availability, followed by a dearth in testing kits and therefore inability to diagnose and treat new cases in the country (Schreiber 2019). Since 2019, only one ART combination has been offered to Venezuelans living with HIV (Acción Solidaria 2019), and this particular pharmaceutical may not be suitable for everyone. Perhaps unsurprisingly, Hopkins (2023) has estimated that as of 2023, only 10 percent of Venezuelans living with HIV within Venezuela are actually taking ART. In addition, a lack of barrier method contraception availability means that people are unable to protect against any STIs (Irons 2022). There is no Pre-exposure prophylaxis (PrEP) available in the country.

The situation is troublesome – Venezuelans living with known HIV cannot access medication, and those living with unknown HIV and/or without HIV are unable to protect against transmission. Some scholars suggest that, due to the developments in treatment, HIV may now be considered a chronic disease rather than a death sentence (Deeks et al. 2013). However, one could argue that this overlooks subaltern realities and only holds relevance for (Global North) contexts where ART is widely available – for Venezuelans, it is important to underscore the fact that neither HIV nor AIDS is death sentences by themselves, however they can become so if appropriate health care access is not guaranteed.

There are numerous reasons why Venezuelans chose to leave the country, with health care being one of the key motivations. As the immediate neighbor and largest host country, as of 2024, there are an estimated 2.8 million Venezuelans in Colombia (IOM 2024). With 2,000 of those living in Colombia estimated to have HIV, as of 2018. Health care through the public health system is not offered free-of-charge to migrants. To access the system, one needs to purchase insurance and belong to a health agent “EPS” (*“Entidad promotora de salud”*). That said, there is also a system of subsided health insurance that supports low-income individuals with free-of-charge health care. However, this is only possible for those holding residency and so is not an option for many Venezuelans (Rodríguez Vargas and López Jaramillo 2021).

Within the Colombian health care system, ART has been available for over 30 years (Machado-Alba and Vidal 2012). However, as Ruiz-Sánchez notes, it is still considered a “gay disease” (Ruiz-Sánchez 2024:216) in the country. There is still a great deal of sexuality-related stigma (Quevedo-Gómez et al. 2012), and as Abadía-Barrero (2004) argues, the rightist government policies at the time of the epidemic outbreak led to significant health care gaps. For example, Colombia’s first case of HIV had been diagnosed in 1983 in the Caribbean coastal city of Cartagena, but ART would not be available until almost a decade later in 1992 (Machado-Alba and Vidal 2012).

Nowadays, for migrants to access ART and associated services such as HIV testing, they need to go to an NGO, of which there are various in the country that provide mediation and support free-of-charge to migrants. As such, the pursuit of the ART that they cannot obtain in Venezuela may be one of the many reasons why people migrate (Irons 2024). According to one study conducted with Venezuelans in Colombia, migrants with irregular legal status were the most likely to have higher HIV viral loads (Wirtz et al. 2023), suggesting a reduced engagement with the care continuum for those without permission to remain. Yet, it should also be noted that there have been efforts on the part of Colombian migration and the health system to support those migrants with temporary permissions in the country (Agarwal-Harding et al. 2024).

Of note, a lack of ART availability does not equate death, and nor is this a predetermined outcome of untreated HIV. For example, a person’s condition may not progress to late-stage HIV/AIDS for up to 10 years following transmission and lack of treatment. At the point at which a person has identified late-stage HIV/AIDS, they may still have the opportunity for successful treatment for another 3–4 years before death occurs (UNAIDS 2024). It is important to underscore this information and the fact that neither HIV nor AIDS is death sentences.

There is a great deal of literature on the experience of HIV/AIDS and structural conditions within the medical anthropology literature, and perspectives from Latin America offer important contributions to this topic. For example, in her study of HIV in Bolivia, Heckert (2016) explores how structural issues are frequently blamed for the poor health outcomes of patients living with HIV. However, she suggests that symbolic violence through medical negligence perpetuates the care of marginalized individuals, and needs to be considered alongside the wider structural challenges. Taking a view from Peru, Perez (2022) explores how gay and transgender communities experienced the global “end of AIDS” declarations against a backdrop of civil violence. They argue that the communities dramatize stories of transactional sex through romanticized gift-giving, which underscores the moralizing nature of “ending AIDS” in Latin America. Finally, and of most significance to the present work, is Brazilian anthropologist Biehl’s (2013) exploration of the refuge home “Vita” – a place for those abandoned by their families due to HIV (and other motivations). In this work, Biehl explores the ways in which those who are abandoned experience social erasure and social death – whilst physically remaining vital. Biehl discusses the rise of zones of *social abandonment* of those who are thought to have been discarded by the state – much like those I will discuss in this article. Here, I will introduce a similar refuge network for people living with HIV; however, the deaths referred to here are objective and not symbolic.

In discussing ghosts of the HIV deceased, I am essentially talking about people who have died of late-stage HIV/AIDS, and though their individual cases are not known, I wish to highlight that death is not a given for all or even most of the Venezuelans and Colombians living with HIV that pass though Santa Chiquinquirá refuge network. The *espíritus* mentioned here are seen and analyzed from the perspective of the living, not as a forbearance of migrant trajectories or predetermined health outcomes. They are thought to haunt, but not to foretell futures.

Anthropology is no stranger to the discussion of hauntings, whether these are framed as ghosts, spirits, or otherwise (e.g. see *Anthropology News* “Ghosts” Special Issue 2021). Ghostly apparitions and/or spirits appear in ethnographies from across the world, from Asia (Shepherd 2019) to Oceania (Murphy 2018), to Europe (Hanks 2016), Africa (Victor 2018), North America (Baker and Bader 2014), and Latin America (Shepard 2002). Santa Chiquinquirá’s beyond-the-living residents may be considered to be “betwixt and between” (Child 2023:119) as ghosts often are; beings in a state of liminality who are neither living nor fully dead. In the Latin American context, they may be considered as souls in purgatory (Klaufus 2019), but either way, they are beyond-the-living and not yet gone from this world. This is a not uncommon perspective in the Global South. As Child notes, “Western societies tend to view death as happening at one time […] however, many other societies regard death as a process” (2023:119).

This process may mean that the dead continue to coexist with the living, a concept that is particularly pronounced across Latin America. Probably the most well-known example of this is All Souls Day, popularized as “Day of the Dead,” whereby deceased humans return to earth to visit the living in the form of spirits (Bryant 2003). Though commonly associated with Mexico, All Souls Day beliefs are found across the Roman Catholic world, including in Colombia and Venezuela. As noted by Marchi (2013), the ritual is a fusion of Catholic and Indigenous beliefs about acknowledging and honoring the dead and is expressed differentially depending on the histories of each country and region. Importantly, spirits do return to the earth again following the expiry of their fleshy vessels, interacting bilaterally with the world of the living.

In literature, such scenarios may be referred to as instances of magic realism, a term of particular cultural importance for Colombia, which refers to “a genuine, spontaneous extraordinary event or even an object often found in daily life across Latin American cultures” (Rave 2003:2).

Ghosts are a common feature in magical realist literature, with specific representation. Hart argues that “the phantom in magical realist fiction is the projection within an ideologically riven nation of a subaltern forced to ‘disappear’ as a result of lying (in both senses of the term) on the wrong side of the political, gender, or race line” (Hart 2003:115). This is an important point of consideration in the story of the Santa Chiquinquirá *espíritus*, who would have been socially marginalized due to the HIV/AIDS and potentially their sexuality, when alive.

Whilst magical realism is often used to refer to a literary genre, when it comes to Colombia it goes beyond this definition. From 2013 to 2023, the country’s official tourism slogan was *“Colombia is magical realism”*. Such nation branding has sought to sell the country to tourists, reinventing Colombia as a “magical place” that is worthy of visiting rather than a violent region prone to attacks and insecurity (Sanin 2017). That said, it is important to note that such country branding may not be supported by the whole population.

Magical realism has been considered as a decolonial literary genre, an “allegory of colonialism” (Simon 2003). In this case, it could better be considered as an allegory of the colonial experience. As such, in line with the beliefs of the people about whom this article is written, and the work of Global South scholars from the region noted above, this work takes ghosts and *espíritus* as objective facts, as beings with whom other beings can and do interact, and with whom they may form biosociality.

## Methods

This article is based on long-term ethnographic fieldwork with Venezuelans and Colombians living with HIV in Bogotá, carried out from 2022 to 2024. Participant observation was carried out in international and local HIV NGOs, including Santa Chiquinquirá.’

Santa Chiquinquirá Foundation has a network of refuge houses that host individuals of any nationality living with HIV/AIDS, and family members where caring responsibilities exist, free-of-charge. The refuges provide shared accommodations, simple daily meals, and visits from health staff in the associated clinic. Two of these refuges are in the Bogotá district Ciudad Bolívar – refuge 1 and refuge 2. In order to maintain anonymity, I have changed details about the refuge houses. However, the historical re-telling of events remains true to the information reported to me. I admit that there are many important details missing regarding the past residents of the refuges: I do not have data about the quantity, nationality, sexuality, gender, length of stay, or any other demographic and social pointers that would be helpful. I do not know how many died in the refuges or of what cause. However, it may also be said that just as important as the objective “facts,” are what present-day residents think and say about past residents/present-day *espíritus*. For that reason, I note where missing data would have been interesting but do not dwell on it. All names are pseudonyms.

In addition to extensive participant observation, 40 testimonies of migrants living with HIV and/or utilizing the services of HIV-related NGOs have been recorded, though not all of these individuals passed through Santa Chiquinquirá or had relationships with *espíritus*. Of the interviewees, the majority of the participants came from the Venezuelan capital of Caracas, followed by Zulia state, Carabobo, Trujillo, Anzoátegui, Merida, Sucre, Guyana, Aragua, Bolivar, Nueva Esparta, and Lara.

I refer to those beyond-the-living in Santa Chiquinquirá as *espíritus* because this is the name given to them by the residents. There is some ambiguity in this term as it may be applied to refer to the ghosts/souls of deceased people that once lived, or it may be used to refer to beings that were never considered to have lived, such as practiced in Venezuelan *santería* (Ferrándiz Martín 2003). From the context surrounding interactions and sightings and the way residents spoke about them, it is understood that the *espíritus* were thought to have once lived – sometimes specific *espíritus* were linked to identified deceased people.

Finally, my position is as a Spanish-fluent, white British woman living in Latin America. As such, I consider it important to acknowledge the necessity for dialogue and consultation with other scholars and wide stakeholders in and from the communities being addressed, as suggested by Savolainen et al. (2023) who argue that it is not the researcher’s biography that is the most important, but their collaborative nature and integrity in their work.

### House of the spirits

Flecks of powdery white plaster stick to my palm as I run it across the smooth, newly finished walls inside Santa Chiquinquirá’s recently re-opened original refuge house 1. “Don’t worry about the dust, *mamita*,” the refuge’s live-in host Jaime assures me when he notices my hand is coated from where I’ve been feeling the interior, “they still need to finish painting.” This space, destined to be a dorm-style bedroom for six or more future residents living with HIV, is flooded with natural light from the large windows looking out to the road below. The final flourish of curtains for privacy and darkness are yet to come – there are no rails just yet. I walk around a little, politely showing my appreciation for this offer to tour the refabrications that have been ongoing to the until-recently dilapidated “original” refuge house 1 owned by the foundation.

Knowing there is a lot to get through whilst daylight remains, Jaime gestures for us to hurriedly move on with the visit as he quickly opens one door or another – this will be a bathroom, here a storage cupboard, there another bedroom. The first floor is airy and bright, it looks as new and fresh as it all is, the plaster barely betraying a bump. From here, Jaime carries on our walk, leading me away from the street-facing windows and toward the back of the property. We approach a staircase, and he gestures for me to proceed, our first room seemingly another bedroom, its allocation betrayed by the metal frame of a foldaway bed absconded in the corner. There is no natural light coming into this room though, and suddenly, I don’t feel like touching the walls. “It feels … heavy, doesn’t it?,” Jaime muses out loud, his words seemingly sticking in the gelatinous air.

This was one of the “back rooms” to the refuge – prior to the refurbishment, it had been a bedroom where people would have gone to rest, away from the overbearing light and noises of the street. A room for people with late-stage HIV/AIDS to spend what were likely final days, at a time when ART was not available in Colombia.

I had been anxious at the prospect of finally seeing in person one of the *espíritus* I’d been hearing about for months, so when the second refuge re-opened, I’d agreed to visit before it became filled with the energy of new living residents. Jaime and I said we’d go at night when the *espíritus* would be more likely to appear, a spur-of-the-moment notion suggested by a group of Jaime’s friends over a bottle of rum. However, a couple of nights before our proposed visit, Jaime had been there alone collecting a few things to take to the refuge house II on the same block. That evening, as he pulled the front door shut to lock up, he noticed the dark silhouette of fingers grasp against the brilliant-white, newly plastered walls, and he had frozen a moment. As he watched, a shadowy figure emerged and beckoned him into the darkness. Jaime didn’t think it wise to follow this unknown *espíritu* and vowed never to go back at night, even less by himself, instead seeking the safety of the daylight to visit this property with me 2 days later. I could try to connect with “them” through the walls though, he advised, if I wanted to feel a presence, but I should know the history of this place and who the *espíritus* were before I tried to connect. And importantly, what had happened to them.

Santa Chiquinquirá foundation’s first refuge house was inaugurated in 1987 in the working-class Bogotá district of Ciudad Bolívar – the same year that the United States’ Food and Drug Administration had approved the world’s first ART treatment. Before the widespread availability of ART, HIV mortality was extremely high, with death often occurring from untreatable cases of AIDS (Trickey et al. 2024). For the initial residents of Santa Chiquinquirá, HIV was certainly not a chronic disease, but may have been more likely a death sentence.

In 1980s Colombia, as today, HIV was concentrated amongst men who have sex with men (MSM). In the 80s and 90s, HIV’s strong association with the LGBTQ community “increased discrimination against both groups” (Prachniak-Rincon and de Onís 2016:158), with homophobia already deeply ingrained in paramilitary and armed insurgent movements (Payne 2016; Serrano-Amaya 2017). As Ballvé (2008) reports, living with HIV or indeed being involved with HIV-related work in any way made one a target of violence, including mass execution of “undesirable” populations like those living with HIV, touted as “social cleansing” (2008:1).

Violence was widespread. From 1964 to the signing of the peace agreement in 2016, Colombia experienced an active war. In a context where violence was a palpable risk, MSM living with HIV in 1980s/90s Colombia were an extremely vulnerable group, both due to the possibility of violence but also due to the social marginalization that may have seen them expulsed from family homes. In fact, until today, Colombian youth report rejection from family members if and when their sexual orientation is revealed (Pineda et al. 2023).

The situation of violence and LGBTQ stigma was especially pronounced in the late 80s, resulting in the necessary formation of refuges where those living with HIV could be housed. Historically, the refuges were palliative – places for individuals socially marginalized due to their sexual orientation, suffering with an untreatable and highly stigmatized condition, to spend their final days in the company of those who understood and empathized. The historic framing of the refuge house echoes the zones of social abandonment described by Biehl (2013); places where an *abandonado* person becomes a so-called *ex-human* caught in a machine of social death, or in this case, physical death as well. What is key here, as will later be discussed, is that the historic residents may not have died in the sense of no longer existing on this plane. That notion complicates Biehl’s concept of the *ex-human*, to which I will return.

AIDS is not a disease in itself, but the absence of effective immune response that may result in patients acquiring opportunistic infections that lead to mortality. As such, not every early resident of Santa Chiquinquirá would have had the same experience in the house nor the same length of stay or treatment/care needs. Nevertheless, all residents lived together, slept together in gender-segregated dorms, ate meals together, and spent their free time together. There may have been residents that survived and moved on from the refuge, but due to a lack of available data, I could not ascertain this and so could not collect information about the experience of living in the refuge in the 80s. That said, the general reflection from contemporary employees about the wider social landscape of that time and the information they had from seeing parts of the original refuge house 1 before refurbishment, was that it would have likely been an unpleasant experience of social abandonment. In light of this, it is a particularly cruel part of Santa Chiquinquirá’s history that the refuge 1 was bombed in 1989.

Nicolás, a nurse who worked at a clinic associated with the refuge, explained to me one afternoon why the houses had no signage or logos that would link them to the foundation; there were historic problems with the neighbors, and they thought it best to remain under-the-radar. “Back then, when someone threw that [petrol?] bomb, they didn’t want to share the block with *sidosas* (AIDS-ridden people),” he noted. At that time (and still in the present day, at times), people were unaware and uneducated about HIV/AIDS. Beliefs abounded that HIV could be contagious, passed through contact or via respiratory fluids, and there was widespread fear of contamination, in Colombia as elsewhere. Maybe that’s why “they” bombed the refuge, Nicolás reflected, or “they” just hated the LGBTQ residents housed inside, the culprit was never found and so the motive could never be confirmed. Indeed, it could have been terrorists, but in the absence of concrete proof, present-day residents and employees are left to speculate – a feeling that they noted made the threat of recurrence feel a little more possible. Regardless of who did the act, the heightened sentiment of stigmatization and social isolation was left behind in people’s retelling of the incident, and the *espíritus* of the refuge 1 in particular are thought to have been victims of this event.

In the literature, sightings of ghosts or spirits are often related with violence (Meinert and Whyte 2020:171) and social anxieties (Parkhurst 2014). Meinert and Reynolds Whyte reflect that “this phenomenon always has particular local characteristics” (2020:171), with the spirits’ characters and actions varied depending on those they haunt. For example, Colombian *espíritus* may be perceived as interacting with guerilla and/or paramilitary executions, with heightened accusations of spiritual interference occurring in a landscape of widespread death (Losonczy 2020). In Venezuela, followers of the María Lionza cult believe that they converse with different *espíritus* about everyday matters – including to address violence in the shantytowns (Ferrándiz Martín 2003). As noted, *espíritus* in and from Colombia and Venezuela, in the context the refuges, are *espíritus*-of-place. And sometimes, as those places dictate, the *espíritus* may have been victims of violence when alive. In Bogotá, the forgotten dead, people whose families may have abandoned them in life and death, are considered to be the most powerful (Klaufus 2019:289). This offers an interesting contrast to Biehl’s concept of the *ex-human*; perhaps in life they were abandoned, but in death, this condition of marginalization turns into strength. The forgotten dead are also linked heavily with migration and displacement. From the 1950s to the peace accord, Colombia suffered mass internal displacement, with rural migrants often dying alone in Bogotá as victims of violence. More recently, the migrants in Bogotá are Venezuelans – but also people suffering alone and potentially having experienced serious violence, differentially akin to the history of the refuge attack.

It was the refuge 1 house that was targeted, and though the refurbishment made it impossible to see where the impact had been, I was told that some of the rooms were made uninhabitable. The foundation had left the house as it was for near-on 30 years, whether due to lack of funds or perceptions of residents and neighbors that the property was so-called “cursed,” was unclear. The story changed depending on who told it. Importantly, the history of the attack was strongly felt in both refuge houses – they are on the same block, and when refuge 1 was bombed, the surviving residents were moved to refuge 2, taking their memories of violent discrimination with them. These residents, bombed and rehoused, made up the initial cohort of *espíritus* inhabiting Santa Chiquinquirá. Who could the dark figure have been that was watching him that night 2 days before our visit to the house, but one of the past residents who had died within the house, reasoned Jaime. Dark, and featureless, perhaps because they had lost their humanly details upon impact with a bomb, he speculated. It is an uncomfortable but significant part of the HIV/AIDS and LGBTQ story in Bogotá.

### HIV/AIDS nekros in the morgue

Though the bomb attack necessitated a complete refurbishment of refuge 1, refuge 2 had also undergone some developments over the years to correspond to the changing needs of the space. Every one of the three floors above the ground remained as bedrooms and bathrooms to house the residents – the use had stayed constant throughout the years. However, parts of the ground floor had been entirely remodelled as the needs of the foundation changed. Of particular interest is the kitchen.

Refuge 2 kitchen is a space where three meals a day are prepared and served for upwards of 10 residents and multiple more guests at any given time. The Colombian non-live-in cook Férula works long hours washing, chopping, peeling, marinading, mashing, rinsing, boiling, all while cheerily moving her dancing feet to whatever rhythm she has going on the speakers. The dining space attached to the kitchen is really the life of the house, and here is where many of the conversations about the *espíritus* took place. It is logical that this room be located at the back of the property – one opens the door directly onto an alleyway where food and refuse can be easily removed from the house and disposed of away from the street and prying neighbor eyes, sent away in garbage trucks. For the same practical reasons, this space was once the morgue.

As noted, prior to the rollout of ART, the refuges were predominantly palliative. It follows that, at times, there was a need to store and dispose of the corpses of the deceased. However, in a country where violence has occurred, there are issues of spatial availability within cemeteries under a Colombian context of a “flourishing death industry” (Klaufus 2016:74). Besides, HIV/AIDS corpses may have been complicated matters. Troyer notes that during the 1980s, the US funeral service faced a new challenge when the bodies of those that had died of HIV/AIDS arrived in the morgues. At the time, the HIV/AIDS corpse was considered “biologically dangerous” (2010:48). Because the virus was new and means of transmission were clinically unknown, “HIV/AIDS produced a new kind of dead body” (2010:49); one that “destabilized notions of universal death” (2010:54) as the virus was thought to potentially remain contagious in the HIV/AIDS corpse following death. What emerged from this period were new “technologies of the corpse,” where corpses came to be treated as potentially hazardous. The HIV/AIDS body was categorized as “an abnormal body” that needed technologies (such as cosmetics) “to make the dead person human again” (2010:62). Importantly, Troyer states that the technologies to transform could result in an erasure of historical parts of a deceased person’s [queer] life. In being an HIV/AIDS corpse, however, the body was able to claim some degree of “postmortem agency” through suggesting membership of the LGBTQ community-a detail that could have been erased in the absence of the virus.

I was not able to obtain information on how bodies were treated at Santa Chiquinquirá in the past; however, due to the ambiguity of the HIV/AIDS corpse it makes sense that they were kept in a part of the building where others may not pass frequently. To restate, these were the bodies of individuals who had died in a refuge, away from family who had potentially disowned them and may not have claimed the corpse. Certainly, the majority of those who died in Santa Chiquinquirá during its early years would have lived with social stigma at a time where HIV positive people and members of the LGBTQ community were at risk of violence.

Of note, it is not only Colombians who have died in Santa Chiquinquirá but also Venezuelans living in Bogotá during the COVID-19 pandemic. Lara, a past resident who came back to visit told me how her friend Blanca, a trans woman from Valencia city, had passed away in the refuge when the pandemic hit, presumably from AIDS-related complications and/or a lack of medication availability during that time. The woman had no family in Colombia, and she was not in contact with her family back in Venezuela either. No one claimed her body, and she was buried by the refuge, following what Lara opined was a lonely death. Apparently, Blanca was still living at refuge house 2 by the time I came to visit, albeit in a different form than before. For her part, Lara, also a trans woman, also had no family in Colombia. She had traveled alone from Caracas in 2020 because her ART was no longer available to her in the city, and she had been forced to stop taking it. Upon arrival in Bogotá, Lara had participated in sex work on and off, before coming to the refuge house due to poor health. She now had ART access and was not facing the same fate as her friend Blanca.

Indeed, many passings in Santa Chiquinquirá may not have been happy deaths, by virtue of the nature of the refuges. Férula the cook, Severo, and Nívea, two Caraqueño refuge residents living with HIV, certainly thought this when discussing some of the interactions that had passed with the *espíritus* in the morgue-cum-kitchen.

It was December 2022, and as Severo had already been at the house for 2 months, he’d become accustomed to some of the nighttime activities. Severo came from the Caribbean city of Maracaibo and had previously mentioned to me some family ties to Venezuelan *santería* (spiritism). As such, he was unfazed when he’d heard a clattering of pans coming from the kitchen the evening before but had run downstairs to check on the situation as Férula had shrieked. “It happens now and then, I’m preparing food and *whomp* a pan drops to the floor, or the cutlery moves,” Férula recounted, noting how it gives her more of a jump scare than anything. Nívea was a little surprised, having only arrived a week ago, but was quickly becoming accustomed to the *espíritus*. Just as I had, new residents learned about the beyond-residents through the frequent stories and reflections of those that had been in contact with them; they came to be part of the refuge experience just as the living residents were, and they sometimes had a say in matters. Once, an *espíritu* had launched Férula’s cellphone at her while she was singing along to a tune, causing the music to stop abruptly – “*quizas* they hated your *vallenato* [Colombian folk music]” quipped Severo in response to this story. Férula switched to reggaeton after that, which the *espíritus* seemed to tolerate. Férula was the only person to report objects moving in this way, but she was also the only person to spend extended periods of time in the morgue-cum-kitchen. When the three of them were discussing this incident, and the general unsettling feeling that being in the morgue-cum-kitchen produced, it was universally agreed that this was an area of great sadness for the *espíritus*, resulting in potential bad behavior in order that they be noticed and receive the empathy they did not get when alive.

Those beyond-the-living at Santa Chiquinquirá were part of a globally unfolding pandemic, but it serves to query their deaths. They were not inevitable, then or now, but arguably came about due to a lack of ART availability that would have suppressed their viral loads and supported their health. As noted, the US FDA approved the first ART drug the same year that Santa Chiquinquirá opened (1987), but this was not given to Colombians until 1992 (Machado-Alba and Vidal 2012). Inevitably, this led to the advancement of HIV and the development of AIDS and eventual death where treatment was never provided. For the past lives of the *espíritus*, understanding the wider health system context helps us to understand how and why they may have ended up in Santa Chiquinquirá, and what their experience may have been. In a nutshell, they would not have had access to ART, despite it existing in the world. Their experience would therefore likely have been quite different from say, a person in the Global North, though there is limited speculation that should be provided here. In the present day, Venezuelans living with HIV are also unable to access effective ART in their country, the result of which may be the development of resistance to ART drugs, a lack of treatment futures (Irons 2024), a development of late-stage HIV and in some cases, death.

When it comes to technologies of power that result in death, Cameroonian scholar Mbembe suggests the concept of “necropolitics” (2003). From the Greek *“nekros”* for corpse, it follows that necropower is a politics of death that specifically results in the production of corpses (Troyer 2010:125). For Mbembe, necropolitics is expressed in the state-controlled power in deciding who will die; those whom the state considers “killable.” In a context of coloniality, this power often targets those deemed hierarchically inferior by a Global North-aligned state-those citizens marginalized due to their ethnicity, race, gender, and/or sexuality. Necropower in the HIV/AIDS context works most clearly through stigma. As the previous section showed, Santa Chiquinquirá’s history strongly suggests that the consequences of social stigma from the community was violence, and death. As a zone of social abandonment inhabited by *ex-humans* (Biehl 2013), refuge inhabitants were arguably transformed into the living dead already before their bodies became corpses. This relationship between necropolitics and HIV/AIDS stigma has been observed in many contexts across the world (Atuk 2024; Cazeiro et al. 2021; Tsang 2020). Here, it could be said that Santa Chiquinquirá’s living and beyond-the-living residents were/are subject to necropower that results in state and social stigma and abandonment, to different degrees.

This history also affects the living, who are aware of the recent past experienced by other individuals, not unlike themselves, living with the same condition. Though there have been many medical advancements in HIV treatment over the decades, knowledge of the morgue-cum-kitchen and the understanding that *espíritus* are present and active there, arguably represents a blurring of the past and the present. However, subsequent medical advancements may mean that whereas once the space was for the removal of bodies [life], it is now life-giving through food. A space that is still shared by all residents – living and beyond-the-living. Nevertheless, it serves for living residents to remember the recent past, supporting empathy not only for each other but for those who came before.

### Biosociality beyond the living

Until now, the *espíritus* have been discussed predominately as unidirectional entities – they are witnessed moving around, they beckon, they throw cellphones poltergeist-style, but without interfering in the matters of the house, as residents that were part of a biosocial collective would. That is not always the case, however. They also interfere in house social matters.

In the summer of 2023, a self-proclaimed communist came to stay. Openly supporting Venezuela’s socialist party and labeling oneself a *Chavista* may be a sticky subject for Venezuelans, many of whom may feel they’ve had to migrate out of the country due to socialist policies. Nevertheless, Miguel was quite clear in communicating his unwavering support of the Venezuelan government, and of accusing fellow Venezuelan residents of being unpatriotic if and when they voiced objections. Originally from Caracas, Miguel’s migrant trajectory had first taken him to Mexico to follow a boyfriend who’d dangled the possibility of a visa marriage. When the relationship broke down, Miguel first returned to Caracas for a spell, but when the ART ran out, he decided to try his luck in Bogotá instead. His political viewpoints are not necessarily integral to the *espíritus* reaction to him (though, we cannot objectively know). However, they were an integral component in the how the other Venezuelan residents perceived him. At the time, there were 10 other Venezuelans (Clara’s family of six, a pregnant woman, two MSM, and the mother of one MSM) and 1 Colombian living in the refuge. Things were tense between the residents and Miguel. When I came to visit the house, Miguel was rarely socializing with the other residents, and before long, Salvador and his mother Pancha had accused Miguel of stealing money from their hidden stash. A more serious accusation came when Miguel one day offered to assist Férula in preparing the lunch – a kindness that came seemingly out of the blue. Twenty-three-year-old pregnant Alba from Zulia told me later that in the afternoon, every single person had slept deeply for hours. She laughed remembering how Pancha was passed out on a chair on the roof, but she too had drifted off for hours that day. It was odd, Alba said, because she’d had such trouble with anxiety-related insomnia. The residents unanimously thought that Miguel had slipped something into the food that he’d uncharacteristically offered to help prepare, perhaps so he could do some thieving in perceived peace. Had he been guilty, Miguel might not have banked on being observed. Though Alba had found aspects of it amusing, they all noted that were it true, dosing HIV patients with sleeping pills was risky business and could have comprised their health.

One night a week later, an *espíritu* intervened. Alba had been dosing restlessly on her lower bunk bed when she spotted a figure rise at the other end of the dorm. Bedrooms were gender segregated, but Clara and Pancha were sleeping in separate rooms with their children. Alba was alone.

The weak streetlights danced lightly over the dorm shadows, and she identified the figure as female from the body shape. However, Alba’s attention was abruptly broken with a sudden *whoosh!* She whipped her neck around toward the entrance to the hallway, just in time to see the door whip shut in the face of unwanted nighttime visitor Miguel. The sound of the slamming door woke up others in the house, and Miguel was admonished for trying to enter the female dorm in the middle of the night (to steal? To violate Alba? they speculated). He was asked to leave the refuge soon after for reasons that remained confidential, however the conclusion was shared by all-the residents didn’t trust him, and nor did the *espíritus*. Alba and Clara later speculated whether they had both seen the same *espíritu*-the woman in the photo, but Alba couldn’t be sure. Perhaps it had been anti-Chavista Blanca, Alba reasoned. Either way, this *espíritu* was seen to be joining in the collective life of the refuge, with the understanding that the *espíritu* knew Miguel was compromising the health of those living with HIV.

However, *espíritus* did not only seek to remove people from the refuge, they also brought them in, as my experience demonstrated.

I first visited the house in April 2022, somewhat by accident. I did not know where the HIV refuge houses were, this anonymity also being a key part of their survival considering their history. I had been at an associated clinic undertaking research as part of a wider project on Venezuelans living with HIV. Nicolás the nurse worked between both the clinic and refuge house and took an interest in me when I explained my work to him. He decided to take me to the refuge, where I met and conversed with some of the residents. It was the start of relationships that have lasted over 3 years now; initial interactions that I thought had been sparked due to Nicolás’s interest in supporting my project. However, he later told me that academic interest was not the reason he brought me over.

Months after meeting the residents and hearing about past refuge guests, I expressed my interest in the *espíritus* to Nicolás and told him about the article I was hoping to write. Jaime had previously invited me to spend the night in the female dorm so that I could test out whether or not the *espíritus* would interact with me as well – an invite I’d declined upon realizing that my nerves might not hold out enough for me to spend the night alone with *espíritus*. I admitted being a little disappointed to have never witnessed any *espíritus* in my life, and Nicolás found this all funny – “But Beck, the reason I took you to the house in the first place was because you were accompanied by someone, didn’t you know.” To my knowledge, I had gone alone. Nicolás asked me if I had any male family members that had died, who could have been my accompaniment. I suggested my father, but our descriptions didn’t match, and besides my father had never set foot in Colombia. He concluded, giving me no small degree of goosebumps, that one of the *espíritus* had decided to bring me to the refuge so that I could tell the stories of the house.

Far from unique to Santa Chiquinquirá, the interference of *espíritus* in social matters is common in Bogotá, particularly for marginalized communities. Klaufus notes that for those groups living in insecurity, reliance on social relationships for survival becomes extremely important, including those “relations with agents from alternative realms” (2019:284), which becomes “especially true for the most vulnerable groups of people stigmatized for being morally deviant, such as people from the LGBTQ community” (2019:284). In Santa Chiquinquirá, the *espíritus* act as part of the wider collective, seemingly protecting the residents from further health complications by rejecting and barring the access of a person who appeared to have bad intent. On the other hand, *espíritus* also acted in a guardian capacity when it came to permitting the access of an anthropologist who planned to tell their stories. These are biosocial interactions between and amongst beings existing with HIV/AIDS in the refuge, the only difference being that some of these beings are living and others are beyond-the-living.

## Conclusion

It could be said that Santa Chiquinquirá’s houses are refuges from stigmatization and the consequences of social isolation and policies that have produced this outcome for many, though exactly how this looks has changed over the years. In the past, the refuges were inhabited by Colombians living and dying with HIV and AIDS. This group lived through a time of violence, at times directed toward them, under a State that did not provide medication on time if at all. The houses are now mostly filled with Venezuelans living with HIV; whilst there is still stigma and significant health policy issues in ART access, coupled with possible violence at home, the treatment futures look different when compared to the past. When viewed in this light, the *espíritus* tell us important information about the new inhabitants of the refuges: times have changed for those living with HIV. From their relationships with those beyond-the-living, the living inhabitants become carriers of memory, thereby linking them not only to the historical context of HIV but to memories of social marginalization in Colombia.

Biosociality beyond-the-living in the refuges serves to keep present the memory of the near-past, and the consequences of social isolation and marginalization. Through the ethnography, it is shown that present-day residents notice and talk about the *espíritus* all the time. They are present, felt, seen, and a part of refuge life. Importantly, their stories and images are discussed and reflected upon by contemporary residents, which in turn keeps the memory of the context alive. Many Venezuelans coming to Bogotá may not have been aware of the history, what is/was similar, and what has changed. In encountering Colombian *espíritus*, Venezuelans become part of a biosocial community with locals who had the same condition as them. For their part, the biosocial relationships with the living mean that the *espíritus* can express important experiences and stories that may have gone unheard when they were alive. As such, the consideration of biosociality beyond-the-living can act as an important analytical tool in understanding the experiences of migrants living with HIV and how they come to interpret their situation and futures, as well as the experiences of those who lived HIV stigma during times of violence. Beyond the context discussed here, the new concept of biosociality beyond-the-living could have significant theoretical and epistemological implications within medical anthropology as a field. Until now, it has been assumed that biosociality takes place between living individuals, providing a narrow field of focus and understanding for both anthropologists and those involved in biosocial communities. By extension, biosociality has then be anchored to specific time-spaces, thereby limiting the potential relationships that could be formed. In considering biosociality with those beyond-the-living, existing epistemologies are challenged, our current theoretical understanding of biosociality transformed.
